# External Fixation for Fracture Stabilization of the Sacrum in 15 Dogs

**DOI:** 10.3389/fvets.2023.1222504

**Published:** 2023-10-23

**Authors:** Jose Antonio Flores, Gian Luca Rovesti, Jesus Rodriguez-Quiros

**Affiliations:** ^1^Hospital Veterinario IVC Evidensia Prïvet, Villaviciosa de Odón, Spain; ^2^Clinica Veterinaria M. E. Miller, Cavriago, Italy; ^3^Departamento de Medicina y Cirugía Animal, Facultad de Veterinaria, Universidad Complutense de Madrid, Madrid, Spain

**Keywords:** dog, external fixation, sacrum, fractures, stabilization

## Abstract

This study aimed to evaluate the feasibility, complications, and outcomes of external fixation (EF) for the treatment of sacral fractures in dogs, either as a primary fixation system or as a complementary technique. A total of 15 dogs with sacral fractures were surgically treated using different EF configurations, either as primary or secondary stabilization. The results were evaluated for the extent of fracture reduction, stability during treatment, complications, and bone healing. In most cases, the outcomes were excellent in terms of bone healing, neurological conditions, and pain assessment. The mean bone healing time was 9.45 ± 5.66 weeks. One (6.66%) patient presented a complication due to the technique. In conclusion, the use of EF should be considered for the stabilization of sacral fractures because of its minimal invasiveness, stability, and ease of application.

## 1. Introduction

Sacral fractures in dogs are commonly caused by vehicular trauma (1–7). The sacrum houses nerve roots that play a role in the pelvic, pudendal, and perineal nerves, in addition to the most tail-end segments of the sciatic nerve. The nerves extending to the tail's base are a further extension of the cauda equina (2). These fractures can lead to pain and varying degrees of neurological impingement, which may compromise urination and defecation (1–9). Moreover, they can result in an inability to support one or both pelvic limbs (8). Various types of sacral fractures have been described in the literature for both canine and feline species. These fractures are classified based on two different systems: one based on the longitudinal axis of the sacrum, with the foramina serving as the boundary between axial and abaxial fractures (1), and the other based on the type and location of fracture lines within the sacrum, resulting in five distinct types (2). Surgical intervention is the preferred approach for treating these fractures, particularly when they coincide with other pelvic fractures or cause severe pain (5, 6, 8, 9). Conservative treatment is considered for patients exhibiting mild neurological dysfunction and minor displacement at the fracture site (3, 7, 8).

While plates, screws, and pins in combination with polymethyl methacrylate (PMM) are commonly employed for the surgical treatment of spinal fractures (3, 10–15), the utilization of external fixation (EF) for sacral fractures is comparatively less frequent (7) than techniques such as compression screws (16), locking osteosynthesis plates (3, 8), transiliac wires (4), or pins for lumbosacral transarticular fixation (5). The EF system, initially described for vertebral fractures (17, 18), offers several advantages in terms of safety, simplicity, patient tolerance, and final outcomes (19).

The primary objective of surgical stabilization is to prevent movement at the fracture site, which can impede healing and lead to pain (18, 20). This retrospective study aimed to test the hypothesis that EF provides reliability for the surgical treatment of fractures of the sacrum, as well as to assess the surgical technique, complications, and outcomes of applying EF for the stabilization of sacral fractures in 15 client-owned dogs.

## 2. Methods

**Figure 1:**
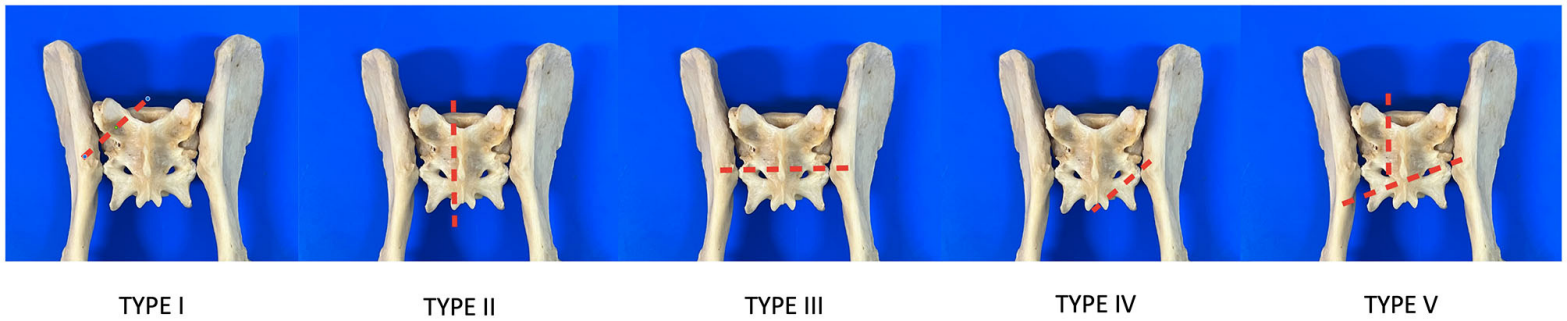
Drawings showing the sacral fractures following the classification described by Anderson et al. (2).

This retrospective study included 15 canine patients. The inclusion criteria consisted of dogs with a diagnosis of sacral fractures treated with external skeletal fixation (ESF). Medical records from Clinica Veterinaria M. E. Miller (Italy) and Hospital Veterinario IVC Evidensia Prïvet (Spain) between 2006 and 2021 were evaluated and included age, weight, etiology, sacral fracture type, presence of additional fractures, surgical treatment, complications, healing time, and clinical and radiographic outcomes. Radiographic controls were conducted by a veterinarian or a radiology technician and scheduled at 3, 6, and 12 weeks post-surgery to validate fracture alignment and the stability of the external fixator during the postoperative time. Additional assessments were conducted as deemed necessary by the surgeon in charge. The fractures were described based on the proposed classification by Anderson et al. (2) (Figure 1):

I. Alar: oblique fracture line on the ventrodorsal radiograph, originating at or immediately adjacent to the juxta-articular notch and terminating at the articular surface of the sacral wing.

II. Foraminal: longitudinal or oblique fracture line on the ventrodorsal radiograph through the first or first and second sacral foramina.

III. Transverse: transverse fracture.

IV. Avulsion: avulsion fracture of the origin of the sacrotuberous ligament.

V. Comminuted: comminuted fracture.

### 2.1. Preoperative management

Preoperative radiographs were taken in latero-lateral and ventro-dorsal projections in sedated patients. The anesthetic protocols were based on medetomidine (100 μg/kg IM; Medetor 1 mg/ml, Virbac, Spain) and midazolam (0.1 mg/kg IM; Midazolam Normon, Laboratorios Normon SA, Spain). For pain management, methadone (0.4 mg/kg IM, Semfortan, Eurovet Animal Health, The Netherlands) was used.

### 2.2. Surgical technique

The canine patients were induced with propofol (2 mg/kg IV, Propofol Lipuro 10 mg/ml; B. Braun Melsungen AG, Germany) and maintained with isofluorane (Isoflo; Ecuphar, Spain) in oxygen. The patients were shaved and disinfected from the proximal area of the tibiae of both pelvic limbs. The position of choice was sternal recumbency with the hind limbs flexed cranially and positioned close to the abdomen (“kneeling dog” position), facilitating lumbosacral joint flexion and fracture reduction. In those with a combination of osteosynthesis plate and ESF, a lateral recumbency was used first, and a sternal recumbency for ESF was adopted later. The surgical technique included two modes of fixation, depending on the patient and fracture characteristics. In general, patients with slight displacement or comminuted fractures were stabilized by EF alone, while those with displaced fractures were stabilized by internal osteosynthesis combined with EF.

The previous approach solely involved external, close stabilization, whereas the latter entailed internal fixation followed by external stabilization. This approach occasionally improved the precision of fracture reduction achievable through the open approach. A stab incision as small as possible was performed, and a sleeve was used as a guide for predrilling and pin insertion. EF frames used 1–3-mm-diameter self-tapping threaded pins, according to the size and weight of the patient, inserted at 300–500 rpm.

**Figure 2:**
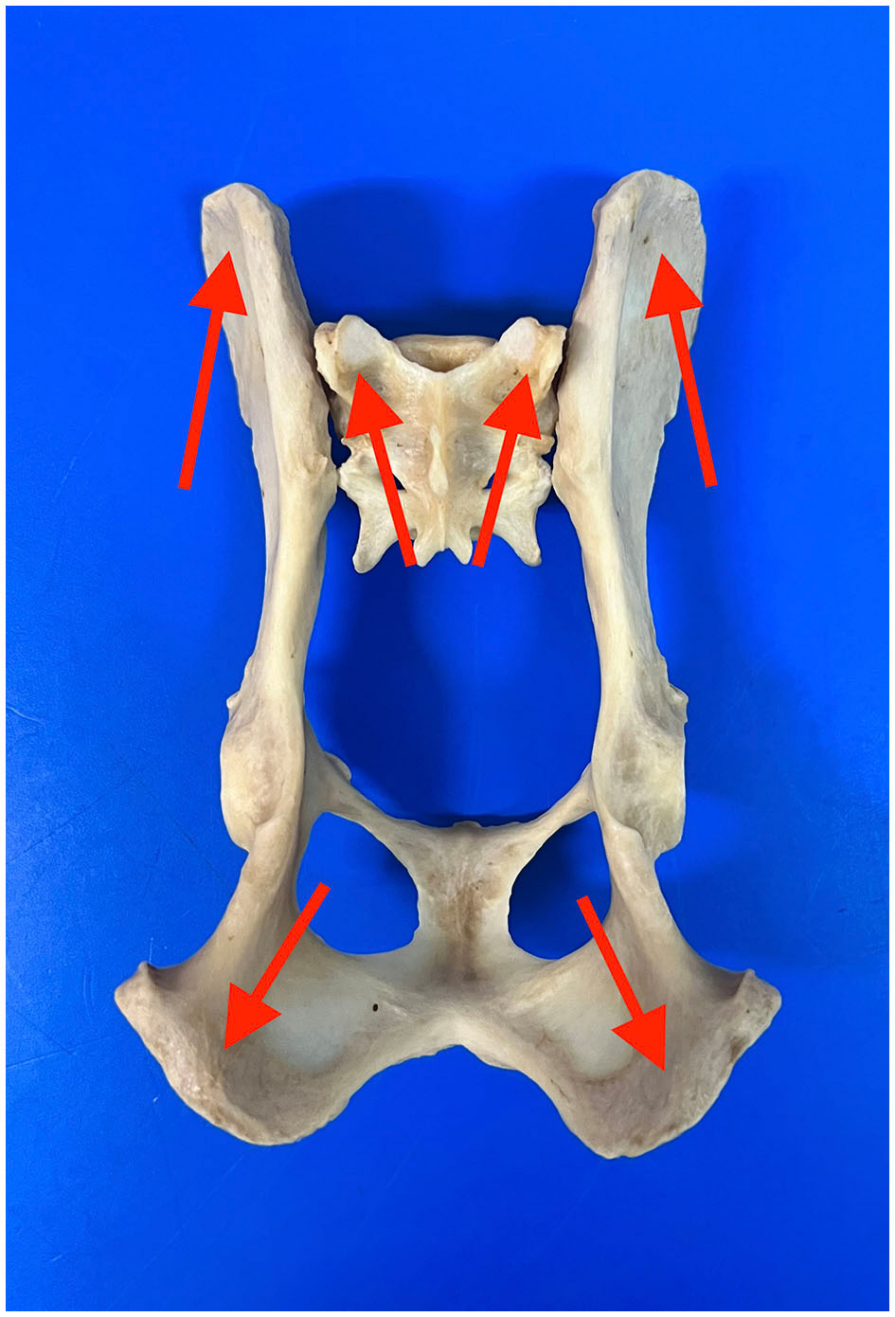
Image showing the locations of the areas for the placement of the pins. Blue arrows: iliac wing; red arrows: ischiatic tuberosity; green arrows: sacral wing.

**Figure 3:**
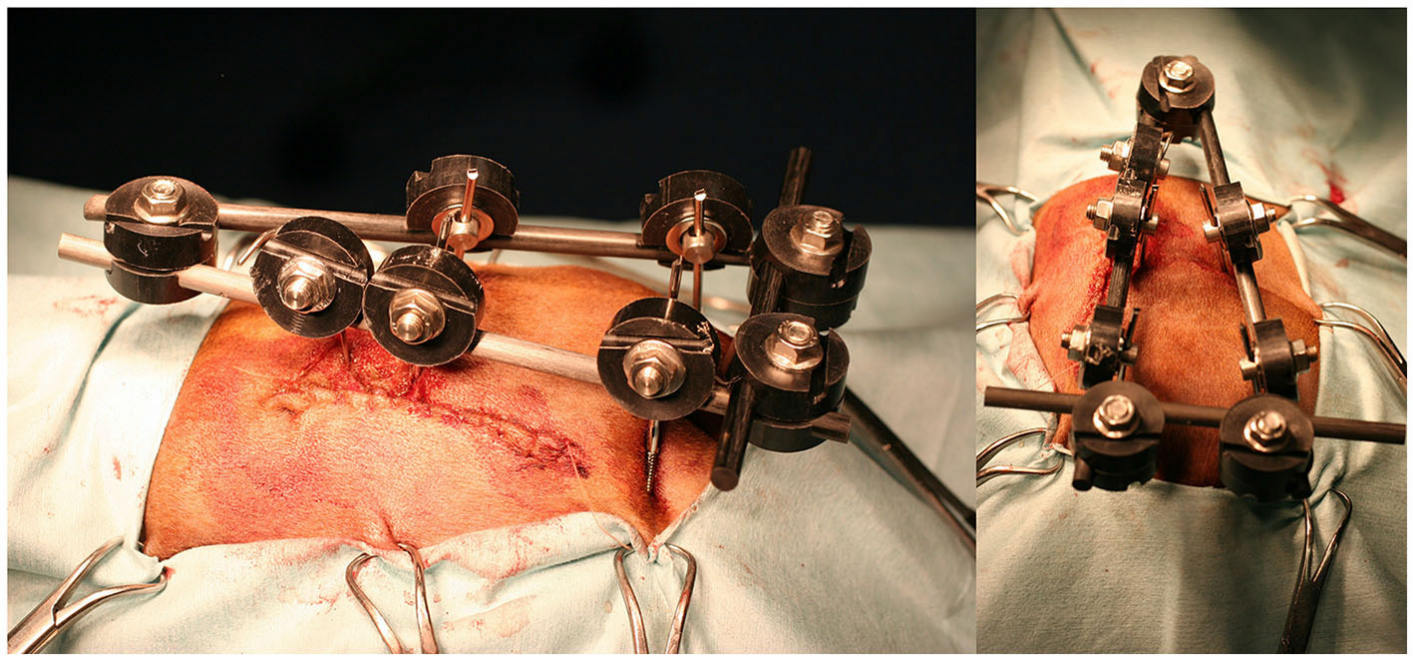
Lateral **(left)** and dorsal **(right)** clinical images of a radiolucent fixator with type O configuration.

**Figure 4:**
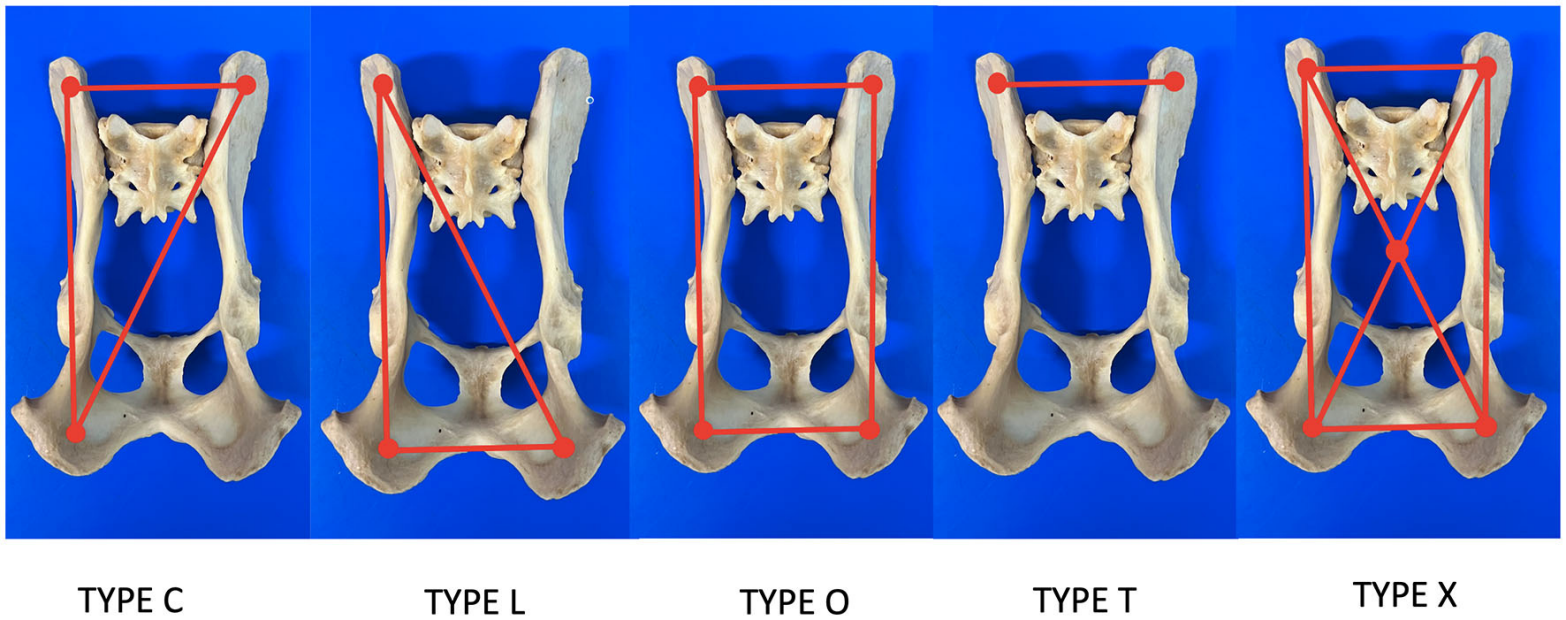
Drawings of the EF configurations used for the stabilization of fractures of the sacrum.

The fracture was reduced using a fluoroscopy-assisted technique, previously locating safe corridors for good placement of the pins (Figure 2). Corridor 1 was located in the sacral tuberosity of the ilium, close to the origin of the gluteus medius. The skin was incised by a stab wound, and the bone is located underlying the fascia. Once the thick dorsal border of the iliac wing was identified, a sleeve was inserted into the wound, the predrilling was performed using an appropriate drill bit, and the pins were inserted through the same sleeve to prevent soft tissue wrapping, at an angle of 10–15 degrees to the vertical, running from proximo-medial to disto-lateral through the same sleeve to avoid soft tissue wrapping. Corridor 2 was located on the ischial tuberosity. A small stab incision was made over the palpable area of the ischial tuberosity. This area is generally located directly lateral to the root of the tail. The wound was enlarged by a small mosquito down to the bone. The technique for predrilling and pin insertion was the same as for iliac pins. The pins were inserted at an entry angle of 10–15° from proximo-lateral to disto-medial. Corridor 3 was located on the sacrum. The reference was located over the median sacrocaudal musculature and the underlying medial crest of the sacrum. A safe site for pin insertion was identified between the median and intermediate ridges, avoiding the dorsal sacral foramina and their paths to the medullary canal. Two other points of insertion were on the sacral wings, in a divergent direction toward the articular processes that articulate the sacrum with the seventh lumbar vertebra. The distraction of the fragments was performed whenever possible after the first pins were inserted, achieving a closed reduction of the fragments by manipulating these pins with a fluoroscopy-assisted technique. The connection of the pins to the connecting bar was performed using Meynard clamps (Insorvet, Barcelona, Spain) and Polilock radiolucent clamps (Ad Maiora, Cavriago, Italy) with 1.2–3-mm steel connecting rods for the Meynard clamps and 5-mm carbon rods for the Polilock clamps (Figure 3). The EF system design was based on individual fracture characteristics, obtaining various configurations based on the involvement of one or both hemipelvises and the type of interconnection between the pins. A review of the types of frame configurations used made it possible to define five types. This classification refers to the manner in which the pins of each hemipelvis are interconnected during the treatment of pelvic fractures with an external fixator (Figure 4). The configurations are the following:

- Type O: The bars interconnect the pins around the perimeter.
- Type X: The bars interconnect the pins on the perimeter with a cross-connection between the clamps located at the vertices of the quadrilateral.
- Type C: The bars connect the pins of one hemipelvis with a pin located on the contralateral ilium or articular sacral process.
- Type T: A single bar interconnects the pins located on both iliac crests and sacral wings.
- Type L: The bars connect the pins of one hemipelvis to the pin located on the contralateral ischial tuberosity.

### 2.3. Postoperative care

The hospitalization period depended on individual patient needs, including postoperative radiographs, fluid and antibiotic therapy, non-steroidal anti-inflammatory drugs, and analgesics. The care of the pin entry holes was performed by cleaning and dressing with antiseptic-impregnated sponges on a daily basis for the first few days and then as required. The care was the same as for standard external fixators, and the owner received instructions on how to perform it to avoid unnecessary patient movement. Strict rest and confinement were recommended during the first four postoperative weeks. Walking on a leash and activity control were recommended until the removal of the fixator. All patients were treated with meloxicam (0.1 mg/kg/24 h PO, Metacam, Boehringer Ingelheim, Germany) for 14 days and gabapentin (15 mg/kg/8 h, Kern Pharma, Spain) for 30 days.

### 2.4. Follow-up

Physical examinations were conducted weekly during the postoperative period, and radiographic bone consolidation of the fractures was assessed if this was the case. In the postoperative period, most controls were performed in lateral projection, and if sagittal projection was needed, ventral recumbency was mandatory due to the presence of the ESF frame.

A functional outcome scale and an EF stability and fracture site displacement scale were used for evaluation. Once radiographic bone consolidation of the fractures was achieved, the external fixator was removed under sedation. Usually, the x-ray controls were 3, 6, and 12 weeks after surgery, although this protocol was changed according to the specific evolution of each patient. The disappearance of the radiographic fracture line was considered complete bone healing, although in some specific cases, a CT scan was performed for better evaluation. Unfortunately, it was not always available, and some owners could not afford it. In those cases, only a radiographic assessment was performed.

A neurofunctional assessment scale regarding the condition of the basic neurological and orthopedic functions at presentation is included. In most of those patients, a standard neurological and orthopedic examination was not possible due to pain, inability to move or stand, and aggressive responses due to manipulations. For these reasons, a simplified evaluation scale was used, which is given as follows:

**Ability to stand**

- No = 0
- One limb = 1
- Two limbs = 2

**Ability to walk**

- No = 0
- With assistance = 1
- Yes = 2

**Ability to urinate spontaneously**

- No = 0
- Occasionally = 1
- Yes = 2

**Presence of pain**

- Severe = 0
- Moderate = 1
- No = 2

**Deep pain in pelvic limbs**

- No = 0
- Doubtful = 1
- Yes = 2

The same assessment scale was applied at the end of the treatment and when further rechecks were scheduled. The score at presentation and the one at outcome were compared.

A third assessment scale was added based on ESF stability and fracture site displacement. Each scale level was assigned a numerical value, which is given as follows:

- Excellent (5): Stable fixator frame with unaltered fracture reduction throughout the healing process.
- Very good (4): Minor signs of instability in the fixator frame or slight fracture displacement. Only minor adjustments to the frame were required without general anesthesia.
- Good (3): Moderate signs of instability in the fixator frame or moderate fracture displacement that did not require surgical revision but required adjustments of the frame under general anesthesia.
- Fair (2): Major instability in the fixator frame or major fracture displacement that requires surgical revision and major adjustments or changes in the fixator frame.
- Poor (1): Major changes in the fixator frame or major fracture displacement that required fixator removal.

## 3. Results

Fifteen dogs with sacral fractures were included in this study. Information related to the breed, age, weight of each patient, date, cause of the trauma, type of sacral fracture, presence of other concomitant fractures, description of the EF configuration used, complications registered, healing time, and final functional results were obtained.

The mean age of the patients included in the study was 4.44 ± 3.76 years (median, 3.5 years; range, 0.3–11 years), and the mean weight was 19.37 ± 12.76 kg (median, 12.5 kg; range, 3–45 kg). The identified causes of the fractures were as follows: run-over (*n* = 11, 73.33%), fall from a height (*n* = 1, 6.66%), and unknown causes (*n* = 3, 20.00%).

In 14 (93.33%) patients, fractures at other locations other than the sacrum were present. The data obtained from the 32 fractures in these 14 patients were as follows: ilium (*n* = 4, 12.5%); acetabulum (*n* = 3, 9.37%); pubis (*n* = 9, 28.12%); ischium (*n* = 9, 28.12%); unilateral sacroiliac dislocation (*n* = 6, 18.75%); and bilateral sacroiliac dislocation (*n* = 1, 3.12%). Extrapelvic lesions were found in one case (6.66%), with bilateral fractures of the femoral head physis, body of the fifth lumbar vertebra (L5), and spinous process of the sixth lumbar vertebra (L6).

According to the classification provided by Anderson et al. (2), the following types of sacral fractures were found: 9 (60%) type I, 4 (26.66%) type III, 1 (6.66%) type IV, and 1 (6.66%) type V.

According to the assessment scale regarding functional and basic neurological conditions at presentation, 14 (93.33%) patients were registered with an inability to walk with their pelvic limbs, and the set of all 15 canine patients in this study obtained an average score of 0.46 ± 0.51 regarding the ability to stand. Severe pain was detected in 14 (93.33%) patients, and an average score of 0.06 ± 0.25 was recorded for this neurological assessment. As for the evaluation of deep pain sensitivity, its presence could not be detected in 2 (6.66%) dogs, and the 15 patients obtained an average score of 1.4 ± 0.38 in this aspect of the scale. The overall mean value for all patients regarding their functionality and neurological records was 0.65 ± 0.38 (median, 0.6; range, 0–1.2). The type of fracture was related to the functional condition, and the data are summarized in Table 1.

**Table 1:** Neurofunctional assessment results regarding the functional and basic neurological status of each patient at presentation, as well as the type of fracture registered in each case.

**Patient no**.	**Fracture type**	**Preoperative ability to stand 0 = no 1 = one limb 2 = two limbs**	**Preoperative ability to walk 0 = no 1 = with help 2 = yes**	**Preoperative ability to urinate spontaneously 0 = no 1 = occasionally 2 = yes**	**Preoperative pain 0 = severe 1 = moderate 2 = no**	**Preoperative perception of deep pain pelvic limbs on neurological exam 0 = no 1 = doubtful 2 = yes**	**Average**
1	I	1	1	1	0	2	1
2	I	0	0	0	0	2	0.4
3	III	0	0	0	0	0	0
4	I	0	0	0	0	1	0.2
5	I	1	0	2	0	2	1
6	I	1	0	0	0	1	0.4
7	III	1	1	0	0	1	0.6
8	I	1	1	1	0	2	1
9	I	0	0	0	0	0	0
10	III	1	1	1	0	2	1
11	I	2	1	1	0	2	1.2
12	V	0	1	1	0	1	0.6
13	III	1	0	0	0	2	0.6
14	IV	1	1	1	0	2	1
15	I	0	0	1	1	2	0.8
AVERAGE	0.66	0.46	0.6	0.06	1.46	

The functional condition of each patient was related to the type of fracture experienced. Patients suffering a type I fracture of the sacrum scored 0.66. Patients with type III scored 0.55, and types IV and V scored 1 and 0.66, respectively (Table 2).

**Table 2:** Neurofunctional assessment score according to the type of fracture at presentation.

**Fracture type**	**No. of patients**	**Neurofunctional assessment score**
I	9 (60%)	0.66
III	4 (26.66%)	0.55
IV	1 (6.66%)	1
V	1 (6.66%)	0.66

Out of the 15 cases treated, EF was used as the sole fixation system in 13 (86.66%). In 2 (13.34%) patients, the EF applied to stabilize the sacrum was combined with two osteosynthesis plates. Of the 13 patients treated with EF exclusively, 8 (61.53%) suffered type I, 3 (20%) type III, 1 (7.69%) type IV, and 1 (7.69%) type V fracture. Of the two patients in whom EF was combined with an internal fixation system, one suffered type I and one type III fracture (Table 3).

**Table 3:** Summary of total fractures classified according to type and surgical technique performed in each case.

**Fracture type**	**No. of patients**	**ESF exclusively**	**ESF and internal osteosynthesis**
I	9 (60%)	8 (88.88%)	1 (11.12%)
III	4 (26.66%)	3 (75%)	1 (25%)
IV	1 (6.66%)	1 (100%)	-
V	1 (6.66%)	1 (100%)	-

Regarding the type of frame configuration of the EF, out of the 15 patients treated, 4 (26.66%) were treated with type X, 4 (26.66%) with type T, 4 (26.66%) with type O, and 3 (20.00%) with type C. When reviewing the type of configuration according to the type of fracture, nine type I fractures were treated as follows: four (44.44%) with type X, two (22.22%) with type O, two (22.22%) with type T, and one (11.11%) with type C. Out of the four cases registered with type III sacral fracture, one (25.00%) was treated with type O, one (25.00%) with type T, and two (50.00%) with type C. The patient with a type IV fracture was treated with type O, and the patient with a type V fracture was treated with type T (Table 4).

**Table 4:** Summary of the data regarding ESF configurations applied to each type of fracture and values of the assessment scale for each ESF type configuration.

**ESF configuration type**	**ESF assessment scale**	**No. of patients**	**Sacral type fracture**				
			**I**	**II**	**III**	**IV**	**V**
X	1.5 ± 1	4 (26.66%)	4 (100%)	–	–	–	–
T	1.25 ± 0.5	4 (26.66%)	2 (50%)	–	1 (25%)	–	1 (25%)
O	1.5 ± 0.57	4 (26.66%)	2 (50%)	–	1 (25%)	1 (25%)	–
C	1.66 ± 1.15	3 (20%)	1 (33.33%)	–	2 (66.67%)	–	–

According to the assessment scale regarding ESF stability and fracture site displacement, the average value for all the cases evaluated is 1.46 ± 0.74 (median, 1; range, 1–3). The assessment based on the configuration used is the following: type X mean value 1.5 ± 1 (median, 1; range, 1–3), type T 1.25 ± 0.5 (median, 1; range, 1–2), type O 1.5 ± 0.57 (median, 1.5; range, 1–2), and type C 1.66 ± 1.15 (median, 1; range, 1–3). This information is summarized in Table 4.

**Figure 5:**
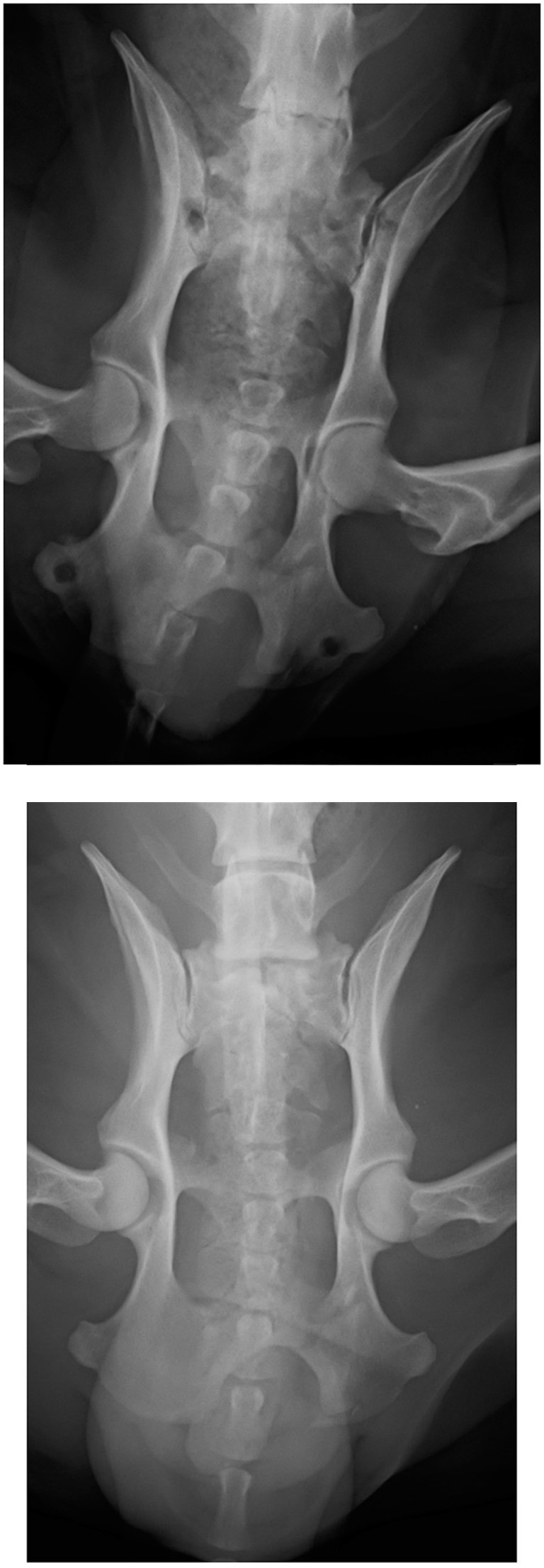
Dorsoventral projection of a type IV fracture at presentation **(top)**. Dorsoventral projection of the same patient 8 weeks postoperatively after ESF removal **(bottom)**.

**Figure 6:**
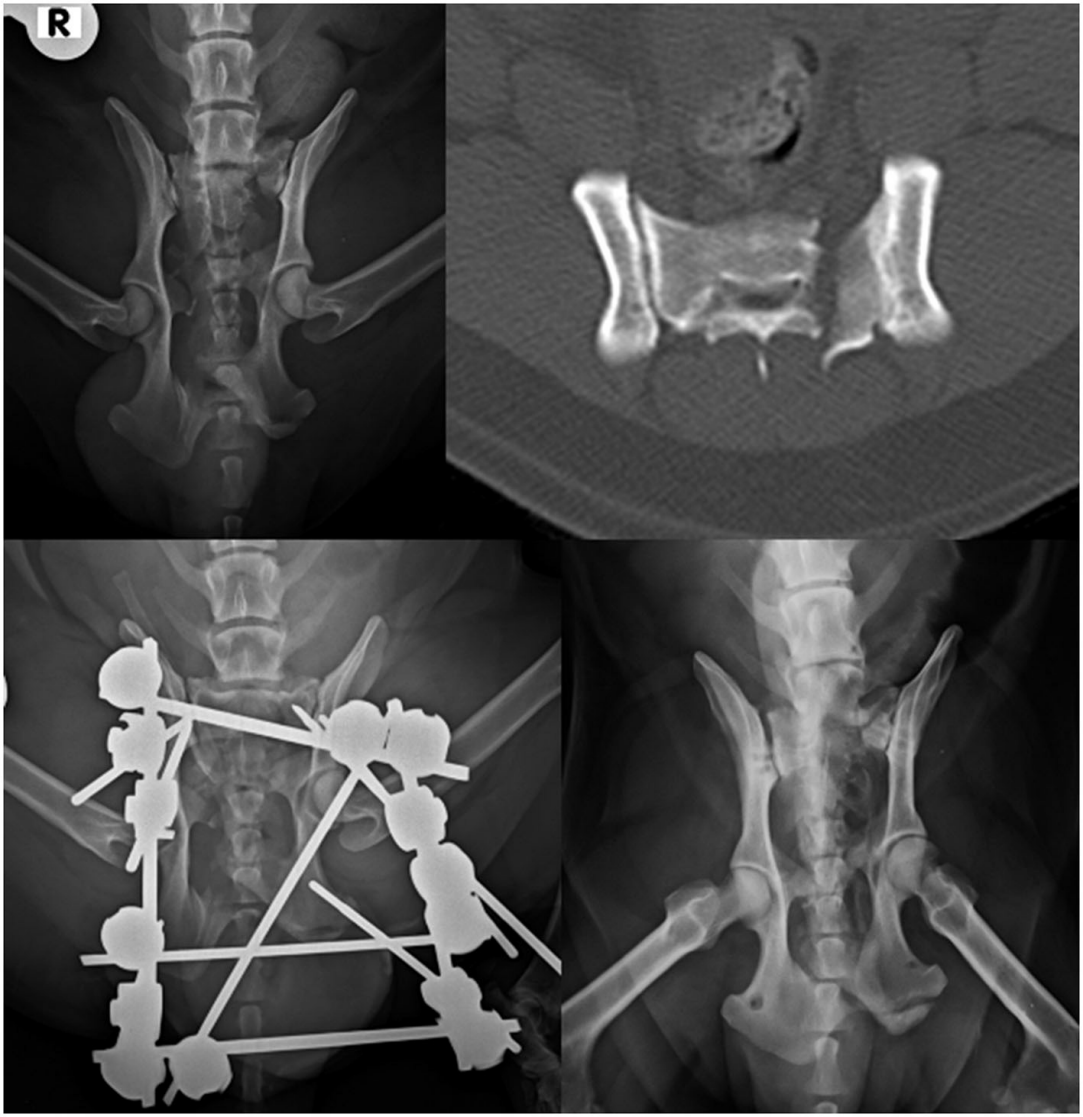
Dorsoventral projection of a type I fracture of the sacrum **(top left)**. CT scan of the fracture **(top right)**. Ventrodorsal projection of the same patient after surgery **(bottom left)**. Follow-up radiograph after ESF removal at 12 weeks **(bottom right)**.

**Figure 7:**
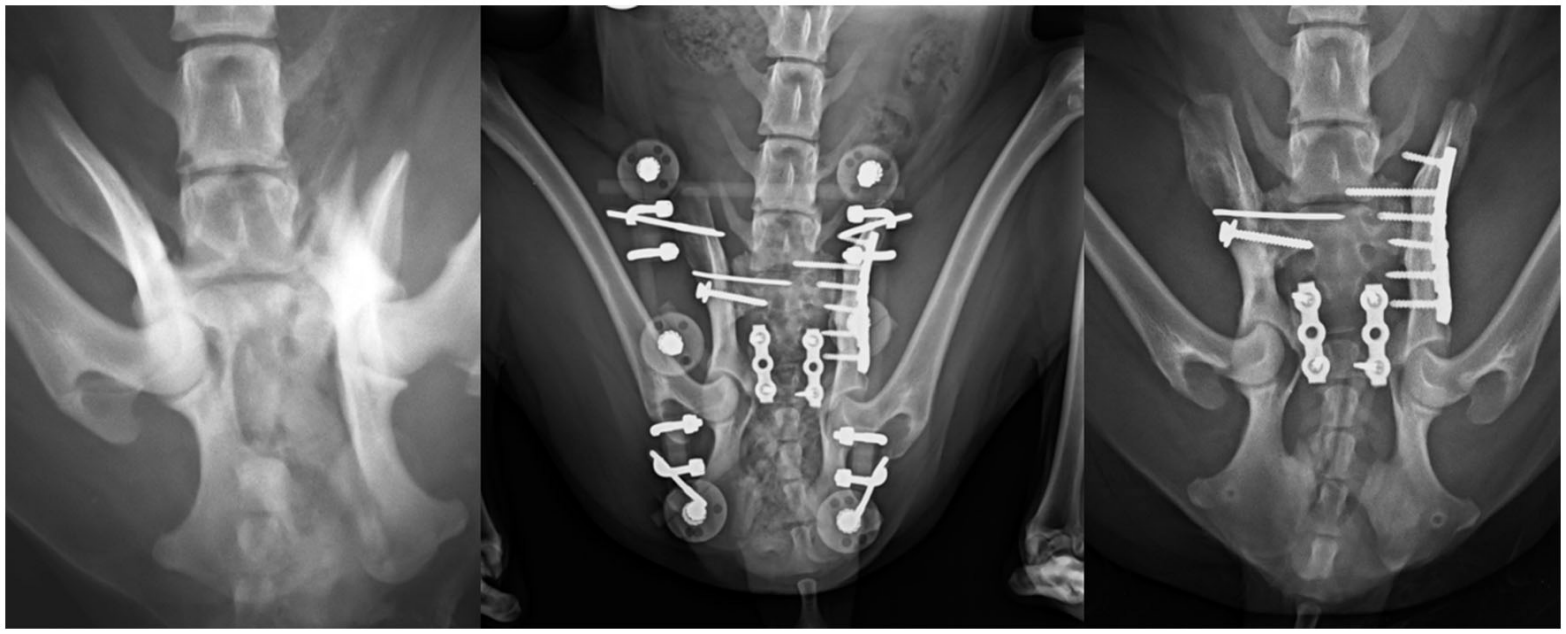
Dorsoventral projections **(left and middle)** of Type III sacral fracture that was treated primarily with internal osteosynthesis by two plates and EF as ancillary stabilization. Note the radiolucent elements of the EF that allowed a better radiographic follow-up. This patient also suffered a left sacroiliac luxation and a fracture of the right ilium. Radiograph after 4 weeks of follow-up when ESF was removed **(right)**.

The global healing time was 9.45 ± 5.66 weeks (median, 8 weeks; range, 2–20 weeks) (Figure 5). According to fracture type, the results are as follows: in eight dogs with type I fracture, the healing time is 10.5 ± 6.45 weeks (median, 8 weeks; range, 2–20 weeks) (Figure 6). In two dogs with type III, it is 6 ± 2.82 weeks (median, 6 weeks; range, 4–8 weeks). In the dogs with types IV and V, it is 5 and 8 weeks, respectively. In the dogs treated with EF in combination with internal osteosynthesis, it is 3 ± 1.41 weeks (median, 3 weeks; range, 2–4 weeks) and 10.3 ± 5.39 weeks (median, 8 weeks; range, 3–20 weeks) (Figure 7). These data are summarized in Table 5.

**Table 5:** Healing time in weeks related to the type of fracture.

**Fracture type**	**No. of patients**	**Healing time (weeks)**	
		**ESF exclusively**	**ESF and internal osteosynthesis**
I	8	10.5 ± 6.45	3 ± 1.41
III	2	6 ± 2.82	10.3 ± 5.39
IV	1	5	–
V	1	8	–

According to the assessment scale regarding functional and basic neurological status at the end of the treatment, once bone healing was achieved and ESF was removed, lameness in one limb persisted in one patient (6.66%). The average rating for this parameter is 1.86 ± 0.55. Another patient (6.66%) presented ambulatory problems that were only possible with assistance. This patient had multiple orthopedic problems as well. Ambulatory capacity had an average score of 1.86 ± 0.35. Out of the 15 patients, 8 (53.33%) had no presence of pain, 7 (46.66%) experienced moderate pain, and 1 (6.66%) had severe pain. The mean pain score was 1.4 ± 0.63. The overall final outcome score was 1.74 ± 0.26. The data are summarized in Table 6.

**Table 6:** Neurofunctional assessment results regarding the functional and basic neurological conditions of each patient at the end of the treatment, once bone healing was achieved and ESF was removed.

**Patient no**.	**Fracture type**	**Frame configuration**	**Postoperative ability to stand 0 = no 1 = one limb 2 = two limbs**	**Postoperative ability to walk 0 = no 1 = with help 2 = yes**	**Postoperative ability to urinate spontaneously 0 = no 1 = occasionally 2 = yes**	**Postoperative pain 0 = severe 1 = moderate 2 = no**	**Postoperative perception of deep pain pelvic limbs on neurological exam 0 = no 1 = doubtful 2 = yes**	**Average**
1	I	X	2	2	2	2	2	**2**
2	I	**T**	2	2	1	1	2	**1.6**
3	III	O	2	2	1	1	2	**1.6**
4	I	X	2	2	2	0	2	**1.6**
5	I	O	2	2	2	2	2	**2**
6	I	O	2	2	1	1	2	**1.6**
7	III	F	2	2	2	1	2	**1.8**
8	I	X	2	2	2	2	2	**2**
9	I	F	0	1	1	1	2	**1**
10	III	T	2	2	2	1	2	**1.8**
11	I	T	2	2	2	2	2	**2**
12	V	T	2	2	2	2	2	**2**
13	III	F	2	2	1	1	2	**1.6**
14	IV	O	2	2	1	2	2	**1.8**
15	I	X	2	1	2	2	2	**1.8**
**Average**			**1.86**	**1.86**	**1.6**	**1.4**	**2**	

The type of fracture and configuration of the frame are included as well.

The complications encountered were mostly of neurological origin due to sacral fractures and their accompanying ones. Some patients experienced more than one kind of complication. Complications were encountered in seven (46.66%) patients and summarized in Table 7.

**Table 7:** Summary of the complications found in the seven affected patients according to the type of fracture and the type of EF system configuration used in each case.

**Patient**	**Type of fracture**	**Type of EF configuration**	**Complication**
2	I	T	Alteration in urination and defecation
3	III	O	Alteration in urination and defecation
4	I	X	Discospondylitis L7-S1
6	I	O	Alteration in urination and defecation
9	I	C	Several complications due to other fractures
13	III	C	Defecation and tail movement
14	IV	O	Alteration in urination and defecation

Of the seven cases with complications, four (57.14%) showed alterations in sphincter function (urination and defecation), which were treated pharmacologically. One (14.28%) had problems moving his tail. In another patient (14.28%), discospondylitis developed at L7-S1, which resulted in chronic pain. In the last patient (14.28%), a large puppy, most complications were due to the simultaneous presence of bilateral pelvic and vertebral fractures.

**Figure 8:**
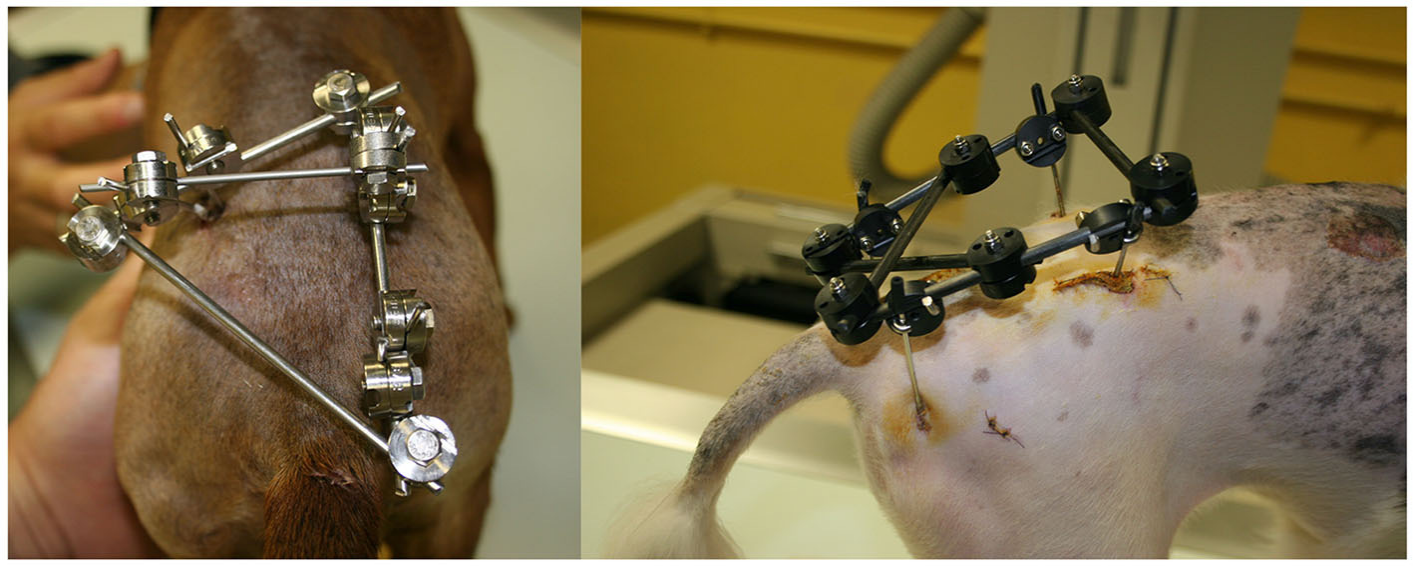
Images of C type **(left)** and X type **(right)** frame configuration.

Of the seven patients that presented complications, four (57.14%) had type I sacral fractures, two (28.57%) type III, and one (14.28%) type IV. Two (28.57%) were treated with EF type C, three (42.85%) with type O, one with type T (14.28%), and one (14.28%) with type X (Figure 8).

## 4. Discussion

To the best of our knowledge, no studies have reported on the use of EF as a stabilization method for sacral fractures in veterinary medicine, unlike in human medicine, where its application has been described as a complementary technique (20, 21). In our research, most sacral fractures resulted from collisions by cars (73.33%), with falls from a height being a less frequent cause (6.66%), which coincides with previous studies (1, 7). These injuries mainly produced type I fractures (60.00%), in contrast to the publication by Anderson et al. (2), in which type III fractures were the most common (50.00%). Falls from a height usually cause transverse sacral fractures, whereas blows from vehicles usually produce longitudinal fractures (2). No type II fractures were present in this case cohort, in accordance with previous studies (1, 2).

Surgical treatment of this type of fracture has been suggested to control the pain and neurological deficits due to the sacrum displacement and impingement on the nerve roots (7). They can be stabilized using plates (8) or pins and screws with PMM (7). The drawbacks of treating fractures of the sacrum are notable due to their anatomical complexity. More data on this subject are available for human medicine, where the difficulty of using fixation systems without causing iatrogenic neurological damage (22) in addition to the instability of most sacral fractures is highlighted (23). In this cohort, 13 (86%) patients were treated with EF only; thus, owing to the minimal invasiveness of the fluoroscopy-assisted technique and closed reduction of the fracture site, the open approach and aggressive manipulation of the fragments were avoided. EF also has characteristics that make its application relatively simple and fast and reduce surgical time, which can be an important issue to consider for the treatment of complex fractures in seriously ill patients (24, 25). In four (26.66%) patients, radiolucent fixators were used which allowed better intraoperative visualization of the bone fragments under fluoroscopic guidance and radiological follow-up. Furthermore, carbon fiber or plastic polymers are lighter than metallic fixators, and this feature can be particularly useful for small dogs.

Regarding ESF stability and fracture site displacement, the T configuration achieved the best grading, with a mean value of 1.25, even though it was applied to complex fractures, such as type III (*n* = 1) and type V (*n* = 1) fractures. In these cases, the complications or delays in bone healing were minimal. The mean removal time for EF was 9.45 weeks, which is considered quite short for this type of fracture.

In two cases, EF was applied as an ancillary stabilization technique together with an internal osteosynthesis technique. In these two patients, EF removal was performed after 3 weeks, whereas for patients treated exclusively with EF, it was 10.3 weeks. In these patients, EF showed a good protective capacity for internal stabilization, preventing its failure and providing a good level of comfort.

Comparing the neurofunctional assessment scale at presentation and at the outcome, a significant improvement was observed after treatment for patients with difficulty in standing up, and only one out of the initial six patients was ultimately unable to stand up spontaneously. The entire group of 15 patients went from an initial score of 0.66 to 1.86 after completing the treatment. This result is similar to previously published studies [Paré et al. (5), Wilson et al. (7)]. Paré et al. highlighted that one (12.50%) patient experienced deficits in a pelvic limb while walking. In our study, two patients scored 1 on our assessment scale, indicating that they needed assistance to walk at the time of discharge (13.32%). The overall score for the 15 cases increased from 0.46 to 1.86, which can be considered a good outcome due to the neurological impact of sacral fractures (5, 7, 8). For the same reasons, it is considered promising that only 1 (6.66%) patient showed residual pain at discharge, compared to the initial 14 patients with severe pain. Similarly to the mentioned references (5, 7), sphincter functionality returned to completely normal levels at the end of the treatment.

These data coincide with bibliographical references that attribute worse results to longitudinal sacral fractures (2), due to their characteristics and greater involvement of the nerve roots.

Mild complications were encountered in seven patients. However, the overall result was considered very good, owing to the frequent iatrogenic damage described and the numerous complications typical of this type of fracture (7).

The retrospective nature of this study is among its limitations due to some technical aspects that changed during the time it was developed, resulting in inconsistent treatment for all cases. Another limitation is due to the complexity of the fractures and the differing circumstances of each patient, which prevented the comparisons between the same conditions. Patients with pelvic and vertebral fractures presented bigger challenges than those with relatively minor trauma. Furthermore, evaluating the biomechanical behavior of the pairing of internal and external systems is difficult. Therefore, specific biomechanical tests should be performed to better clarify the performance of this combination.

## 5. Conclusion

EF is an osteosynthesis technique characterized by its minimal invasivity, relative simplicity of application, and lower cost compared to internal osteosynthesis implants (26, 27). To the best of our knowledge, no previous studies have described the use of EF for sacral fractures in the veterinary literature.

The results obtained in this case-cohort study showed that EF is a good stabilization system for sacral fractures and, owing to its aforementioned characteristics, should be evaluated for the treatment of patients with this kind of lesion. The improvements observed in various aspects of patient wellbeing underscore the potential clinical utility of the technique in addressing the challenges presented by such injuries.

In conclusion, EF showed excellent outcomes in the stabilization of sacral fractures in dogs, with minimal invasiveness and a low complication rate. The combination of internal and external systems provided good protection and comfort to the patients. EF should be considered a primary or complementary technique for internal osteosynthesis, although more studies are needed to gather data on its clinical application.

## Data availability statement

The original contributions presented in the study are included in the article/supplementary material, further inquiries can be directed to the corresponding author.

## Ethics statement

Ethical approval was not required for the studies involving animals in accordance with the local legislation and institutional requirements. It doesn't need ethical approval by an Ethical Committee because all our patients were pets treated with the informed and signed consent of each owner, without testing in any case, since they were not neither experimental nor commercially available animals. Our patients were experimented with at any time. This work is based on the description of surgical techniques already described but modified. Written informed consent was obtained from the owners for the participation of their animals in this study.

## Author contributions

JF, GR, and JR-Q: conceptualization, validation, resources, visualization, and writing—review and editing. GR and JR-Q: methodology and formal analysis, data curation, supervision, and investigation. JF: software and writing—original draft preparation and funding acquisition. JR-Q: project administration. All authors have read and agreed to the published version of the manuscript.
